# Following Transient Conformational Adaptations during Peptide-Guided Calcium Phosphate Biomineralization with Hyperpolarized NMR

**DOI:** 10.1021/jacsau.6c01076

**Published:** 2026-09-16

**Authors:** Fanny Kormout, Ertan Turhan, Mehdi Soussi-Therond, Milan Zachrdla, Dennis Kurzbach

**Affiliations:** † 26909 University of Vienna, Faculty of Chemistry, Institute of Biological Chemistry, Währinger Str. 38, Vienna 1090, Austria; ‡ Doctoral School of Chemistry (DoSChem), Faculty of Chemistry, University of Vienna, Währinger Str. 38, Vienna 1090, Austria

**Keywords:** Hyperpolarized NMR, Dissolution dynamic nuclear polarization, Peptide-guided mineralization, Material formation, Calcium phosphate biomineralization

## Abstract

Peptide-guided biomineralization is a key process involved in the formation of calcium phosphate (CaP) in bone, yet the peptide–ion encounter complexes at the onset of this process have remained structurally invisible: methods with sufficient resolution to decipher these intermediates would already have lost them to precipitation. Here, we resolve how a mineralization peptide adapts its conformation to recruit inorganic ions during the earliest stages of CaP formation. Using a peptide derived from the calcium-binding domain of secreted phosphoprotein 1 (SPP1), a key regulator of bone formation, we find that Ca^2+^ association reorganizes the conformational ensemble toward states with increased solvent exposure of Ca^2+^-coated Asp-rich stretches. These polyionic microenvironments subsequently exhibit spectral perturbations upon phosphate recruitment, consistent with the formation of encounter complexes during the onset of CaP nucleation at the peptide–solvent interface. Access to these transient states is enabled by NMR sensitized with hyperpolarized water (HyperW), which provides a two-order-of-magnitude sensitivity gain and reduces multidimensional acquisition times from hours to seconds—fast enough to capture peptide–ion assemblies immediately after encounter and before precipitation. Together, these results reveal a stepwise mechanism by which an intrinsically disordered mineralization peptide senses and organizes calcium and phosphate ions at the onset of biomineralization.

## Graphical abstract

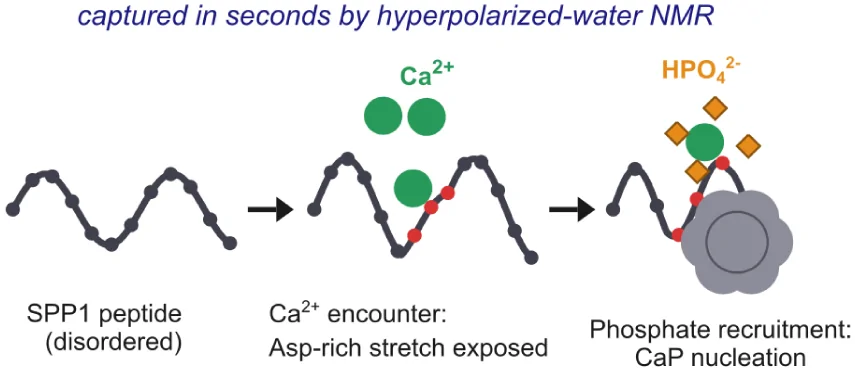

## Introduction

Biomineralization is Life’s strategy to generate highly functional composite materials with remarkable mechanical and structural properties.^1–4^ Examples include bones or teeth whose architectures emerge from an interplay between inorganic ions and structure-guiding biomolecules. Understanding the molecular principles underlying these processes is fundamental, not only to rationalize mineral formation but also to enable biomimetic design of advanced materials.^5–9^

Among the most prominent examples is the formation of calcium phosphate (CaP), the principal mineral constituent of bone.^10–13^ A recurring feature of bone mineralization is the association with highly acidic and often intrinsically disordered proteins (IDPs)^14–16^ that coordinate the inorganic ions and guide mineral formation. This includes the Dentin Matrix Protein 1 (DMP1), the Bone Sialoprotein (BSP), and the secreted phosphoprotein 1 (SPP1, osteopontin). All these molecules have a highly acidic Asp/Glu-rich sequence in common.^6,12,17,18^ However, despite decades of research^19–21^ and the vital nature of bone formation, the molecular basis of these peptides’ modes of action remains largely undisclosed.

SPP1 is a particularly striking example. It is known to bind Ca^2+^ ions, to regulate the nucleation of biological CaP and, thus, to influence the morphology of the resulting mineral phase.^22–24^

Yet, while SPP1’s capacity to bind Ca^2+^ and influence CaP morphology is established, the molecular events occurring in the narrow window between ion encounter and mineral nucleation—the step that actually determines the mineralization pathway—have never been directly observed.^24,25^

This lack of mechanistic understanding is largely a consequence of an experimental bottleneck. The molecular peptide-ion complexes that initiate CaP mineralization are complex, highly dynamic, and transient, existing only briefly before CaP solidification and precipitation.^26,27^ While nuclear magnetic resonance (NMR) spectroscopy is uniquely suited to characterize such disordered biomolecular systems at atomic resolution, conventional NMR lacks the sensitivity to reach these species within their transient lifetime—the very acquisition times needed for residue resolution guarantee that precipitation occurs first. As a result, the molecular events linking calcium coordination, phosphate recognition, and mineral nucleation have not merely been difficult to observe; they have been structurally inaccessible.

Here, we provide a route toward overcoming this experimental bottleneck by employing hyperpolarized water (HyperW) as a signal-enhancing solvent for biomolecular NMR.^28–33^ HyperW boosts NMR sensitivity by more than two orders of magnitude, reducing multidimensional acquisition times from hours to seconds and enabling residue-resolved description of the modes of action of biomineralizing peptides immediately after counterion encounter and, thus, before precipitation as peptide-mineral composites.

This allowed us to capture the molecular state of a Ca^2+^-peptide complex derived from SPP1’s calcium-binding domain (CBD) immediately following phosphate encounter and prior to peptide–CaP co-precipitation. The resulting data support a model in which Ca^2+^ interaction induces a reorganization of the conformational ensemble of the CBD, leading to preferential solvent exposure of Ca^2+^-coated aspartate residues. These Ca^2+^-rich microenvironments form a phosphate-interaction interface, thereby providing a molecular framework to link peptide-calcium binding to the initiation of CaP nucleation. Our results thus provide atomistic insight into molecular-level aspects of SPP1-guided biomineralization while demonstrating how hyperpolarized NMR can access transient molecular states that are inaccessible to conventional structural biology approaches.

## Results and Discussion

Our investigations focused on the isolated CBD of human SPP1 spanning residues 41–90 of the full-length protein (henceforth denoted SPP1^41–90^; Figure 1a). It allowed us to derive residue-resolved mechanistic features associated with SPP1’s biomineralizing activity under controlled in vitro conditions. Thus, our aim is not to resolve the full mechanism of SPP1-guided biomineralization but to demonstrate that hyperpolarized NMR provides access to the transient peptide–ion complexes that guide precipitation.

**Figure 1:**
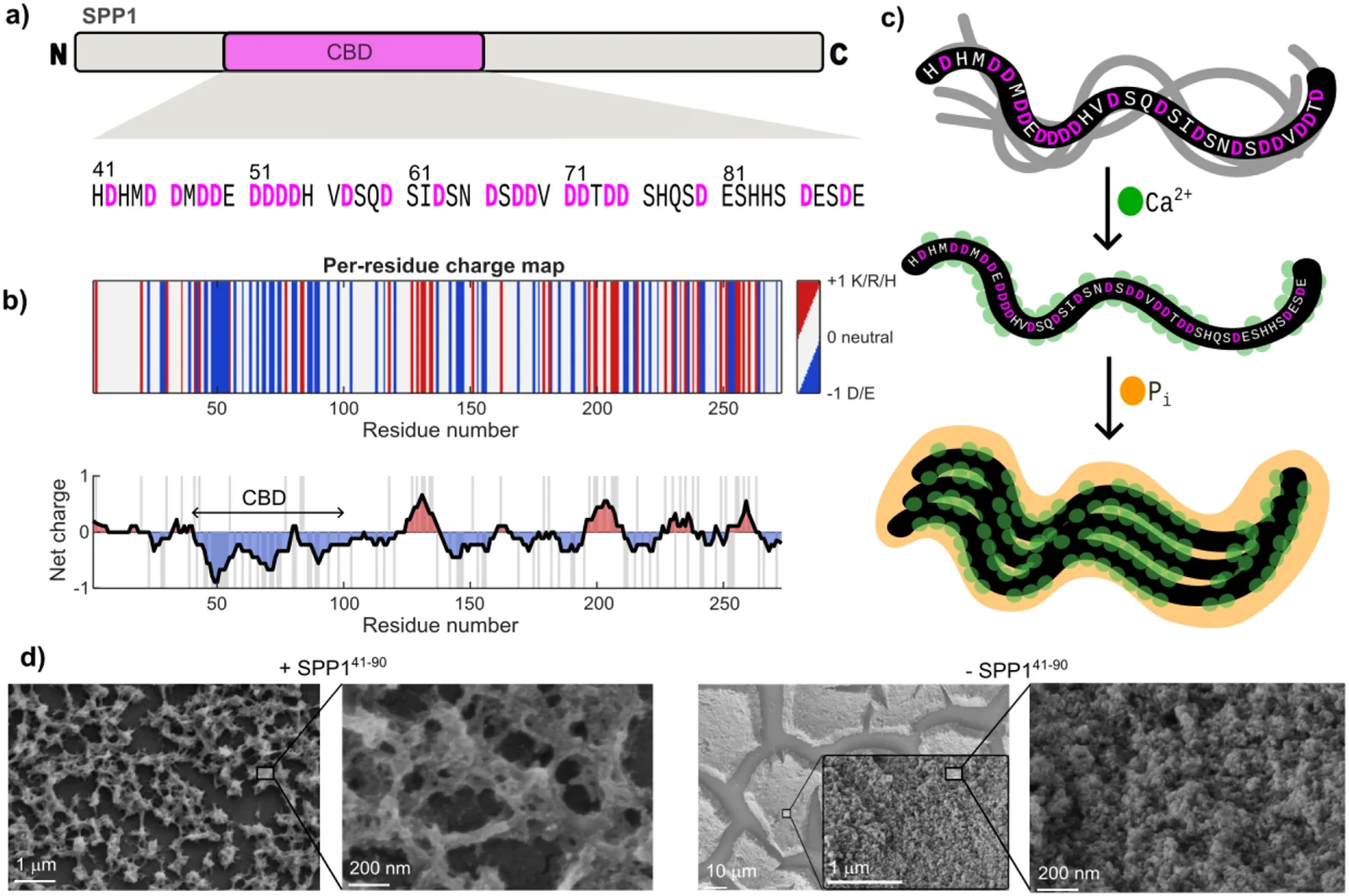
The SPP1-derived peptide system under study. **a)** SPP1 is a ca. 300 amino acid-long intrinsically disordered protein. An aspartate-rich calcium-binding domain (CBD) is centered around residues 40–90. The 50-amino acid-long peptide SPP1^41–90^, housing the negative charge hotspot of the CBD, is the subject of this study. **b)** Distribution of positively (red) and negatively charged (blue) residues (top) and average net charge of SPP1^41–90^ as a function of primary sequence (bottom; moving average over 9 residues). The CBD is highly negatively charged, with the maximum negative charge at residue position 50. **c)** Sketch of the hypothesized function of the CBD in calcium phosphate (CaP) formation. Ca^2+^ ions bind to the CBD, leading to a Ca^2+^-rich microenvironment. Encounter with inorganic phosphate (P_i_) then induces CaP coprecipitation, leading to peptide-guided mineralization with specific solid-phase morphologies. **d)** Scanning electron micrographs of CaP formed in the presence of SPP1^41–90^ (left) and in its absence (right) at different magnifications using 60 eq. of Ca^2+^. The peptide induced the formation of porous CaP structures, distinct from the smooth solid formed in the absence of any peptide. While the impact of SPP1 on CaP formation is thus evidenced, the mechanism underlying its activity remains hypothetical.

SPP1^41–90^ is highly enriched in aspartate residues, 22 in total, that generate a strongly negative electrostatic potential (Figure 1b). Previous studies showed that full-length SPP1 binds Ca^2+^ ions via its CBD and adheres to hydroxyapatite crystallite surfaces.^34–36^ Furthermore, SPP1 was suggested to participate in stabilizing transient calcium phosphate precursor phases (prenucleation clusters) associated with non-classical mineralization pathways by complexing Ca^2+^ ions and kinetically regulating material precipitation.^27^

These observations have led to the hypothesis that Ca^2+^ ions accumulate within the CBD, generating peptide-ion assemblies that can recruit inorganic phosphate (P_i_) from solution to initiate CaP formation under control of the peptide (Figure 1c).

To test this hypothesis experimentally, we first verified that the isolated CBD retains the ability of native SPP1 to influence CaP mineralization. Scanning electron microscopy (SEM) revealed clear differences in the morphology of the CaP solids formed (i) either after incubation of Ca^2+^ with SPP1^41–90^ (Figure 1d) before P_i_-exposure or (ii) through direct mixing of Ca^2+^ and P_i_ solutions. CaP phases formed from Ca^2+^-SPP1 complexes exhibited porous, interconnected networks, while mineralization in the absence of SPP1^41–90^ resulted in smooth surfaces. These observations demonstrate that the isolated CBD preserves an impact on mineral deposition in terms of size and morphology and therefore constitutes a suitable model system for investigating the molecular mechanisms underlying SPP1-guided CaP mineralization.^37^

In vivo, SPP1 is typically highly phosphorylated, particularly at its ca. 30 serine sites,^38^ and the phosphorylation state is known to be a modulator of its mineral-regulatory function. The additional negative phosphate charge is key in Ca^2+^ and, consequently, P_i_ recruitment. Thus, the mineralizing activity reported herein, with a non-phosphorylated SPP1 fragment, should not be considered a physiological process. It isolates the Asp contribution to Ca^2+^ recruitment, and the structure–function relationships we derive should be read as applying to this carboxylate-driven regime only. However, our study primarily aims to demonstrate the capacity of DDNP and HyperW to monitor such transient intermediates in partially biomimetic mineralizing systems and to derive structure-function relationships that allow one to understand the peptide-guided formation processes of biomimetic minerals better.

Next, we characterized the interaction between SPP1^41–90^ and Ca^2+^ (Figure 2a) by conventional solution NMR. This provided the framework for subsequent experiments in HyperW aimed at capturing the transient molecular states that emerge immediately after P_i_ encounter.

**Figure 2:**
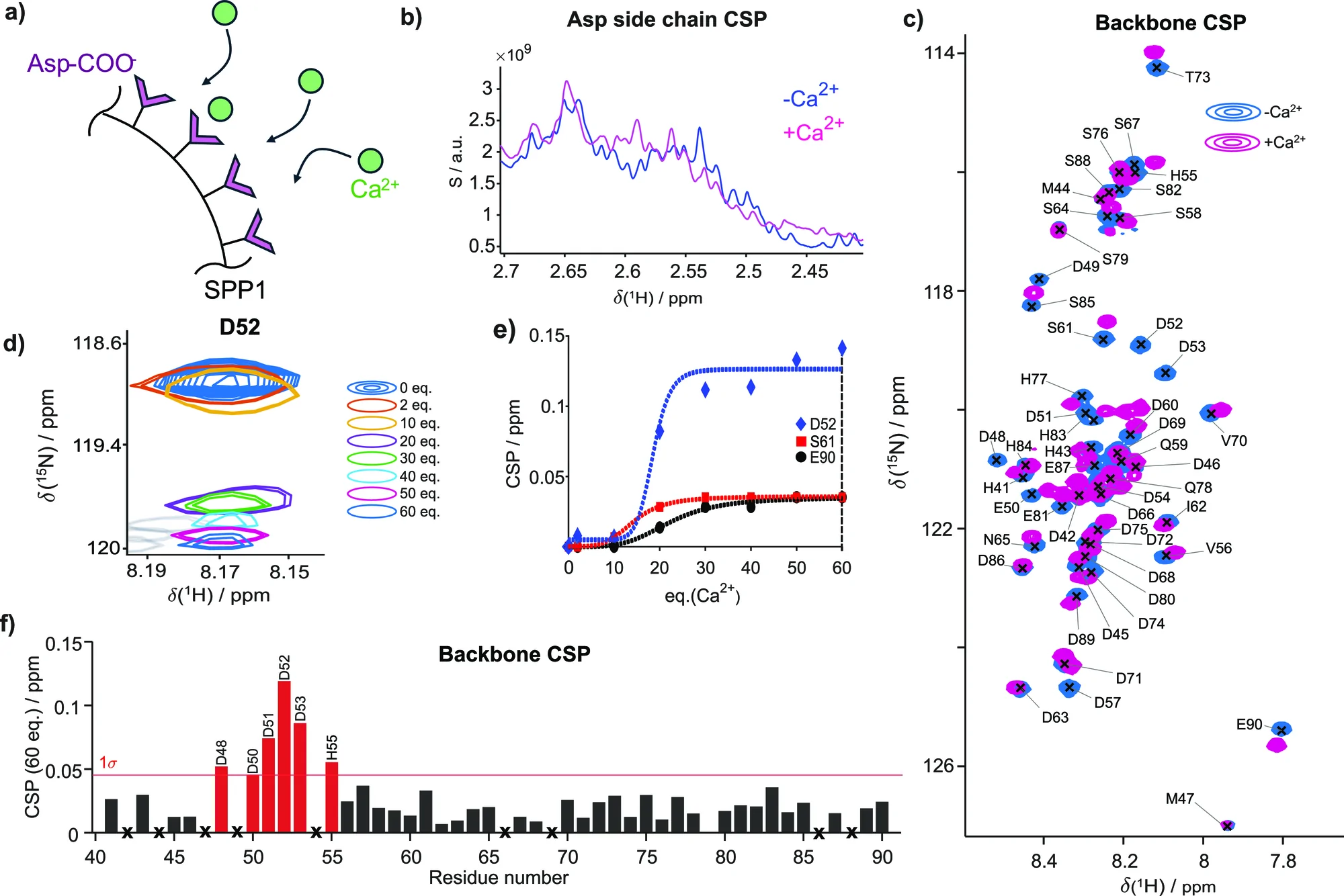
NMR characterization of SPP1^41–90^-Ca^2+^ binding. **a)** Sketch of the system under study: interaction of the Asp-rich domain of SPP1 with Ca^2+^. **b)** ^1^H NMR spectra of Asp H^β^ side-chain resonances in the absence (blue) and presence of Ca^2+^ (magenta). Upon counterion interaction, a multitude of chemical shift perturbations (CSP) can be observed. Due to the strong signal overlap, residue-resolved analysis was not possible. **c)** ^1^H–^15^N HSQC of SPP1^41–90^ backbone amides, in the absence (blue) and presence of Ca^2+^ (magenta). Due to the higher resolution, residue-resolved analysis is possible. The resonance assignment is indicated. CSP can be observed across the entire spectrum. **d)** Zoom on residue D52 showing a strong response to Ca^2+^. Note that increasing Ca^2+^ concentrations entails a sigmoidal response of the chemical shift (little change at low and high Ca^2+^ content, versus great changes at intermediate concentrations). Gray contours correspond to other residues. **e)** Examples of sigmoidal CSP as a function of Ca^2+^ content. Different residues (exemplarily shown D52, S61, E90) react differently, yet at 60 eq. Ca^2+^, corresponding to 2.7 Ca^2+^ ions per Asp, all residues’ titration curves reach a plateau. Hence, this condition waschosen for further analysis. Solid lines represent fits to sigmoidal Hill functions. **f)** Combined ^1^H–^15^N CSP at 60 eq. Ca^2+^. All residues show at least slight changes. Changes with >1σ CSP perturbation (taken over all CSP values) are indicated in red. The strongest reaction was observed between residues 48 and 55. This stretch has the most pronounced negative charge (cf. Figure 1b) and, thus, acts as the primary interaction site for Ca^2+^. Xs indicate nonassigned CSP due to signal overlap.

Exposure of SPP1^41–90^ to Ca^2+^ immediately induced pronounced changes in the ^1^H spectrum, particularly in the Asp side-chain resonances (Figure 2b), indicating the expected binding between the divalent cation and the highly abundant negatively charged COO^–^ moieties. However, due to the substantial spectral overlap of the many Asp resonances, a residue-resolved analysis was not possible.

We therefore turned to two-dimensional ^1^H–^15^N correlation spectroscopy of the peptide backbone. Upon addition of Ca^2+^, chemical shift perturbations (CSPs) were observed throughout the entire spectrum (Figure 2c), i.e., affecting almost all backbone amides and indicating that calcium binding significantly affects the conformational ensemble of the peptide. Inspection of individual residues’ responses to increasing Ca^2+^ concentrations (Figure 2d,e) revealed that some residues reacted strongly, already at low Ca^2+^ concentrations (e.g., D52). Others responded more slowly and with less pronounced CSP (e.g., S61, E90). Despite these different responses, all titration curves (representative examples in Figure 2e) exhibited characteristic sigmoidal behavior and approached saturation at approximately 60 equiv of Ca^2+^ (per peptide; 2.7 Ca^2+^ per Asp). This concentration was therefore chosen for all subsequent experiments to ensure complete occupancy.

Note that such concentrations are far from in vivo conditions. As our study is primarily driven to establish a method capable of capturing transient intermediates in biomimetic mineralization processes, the chosen conditions still constitute a controlled experimental environment, in which no partially populated states complicate the analysis. Indeed, the reported conformation is the end point of Ca^2+^-induced conformational changes rather than the distribution of states populated in vivo.

The sigmoidal nature of the titration profiles observed for both fast- and slow-responding residues suggests that calcium association is not governed by independent binding events but proceeds cooperatively.^39^ Fitting the titration profiles with an empirical Hill model yielded Hill coefficients of n_H_ =9.9, 4.12, and 4.08 for D52, S61, and E90 (all details and spectra in the Supporting Information and Figure S1), consistent with a cooperative response to Ca^2+^ exposure, i.e., progressive Ca^2+^ binding. In context with the ^1^H NMR data (Figure 2b), this indicates the formation of a calcium-rich microenvironment involving the Asp side chains.

The apparent Hill coefficient of 9.9 obtained for D52 should not be interpreted stoichiometrically. The CBD is an intrinsically disordered, polyanionic segment whose Asp carboxylates are not independent binding sites. A possible explanation for the high coefficient is the following: Each bound Ca^2+^ progressively exposes the remaining Asp residues in an arrangement favorable for further coordination. Binding and conformational rearrangement are thus coupled, and the resulting titration curves are far steeper than any model with fixed, independent sites would predict. The Hill coefficient is therefore an empirical measure of the sharpness of this coupled exposure-binding transition rather than a count of bound ions. Besides, DOSY (Supporting Information Figure S2) shows an almost unchanged translational diffusion coefficient across the titration, and the Asp resonances retain full intensity, excluding Ca^2+^-induced oligomerization as the origin of the apparent cooperativity.

To identify the primary Ca^2+^ accumulation site, we next quantified ^1^H–^15^N CSPs at 60 equiv. of Ca^2+^ (see the Supporting Information for details) and mapped them onto the primary sequence (Figure 2f). Although perturbations were detected throughout the entire peptide, the largest responses clustered within residues 48–55. Notably, this region coincides with the most negatively charged segment of the peptide, as identified in Figure 1b. The observed localization of the strongest CSPs therefore confirms that residues around this site are the principal calcium-interaction hotspot within SPP1^41–90^.

Note that full-length SPP1 tends to aggregate under some buffer conditions in the presence of Ca^2+^.^34^ This is yet not the case under the present conditions, as confirmed by diffusion-ordered spectroscopy that showed only a slight decrease in diffusion coefficient from 2.2 to 1.9·10^–10^ m^2^s^–1^ upon Ca^2+^ coordination (Supporting Information Figure S2).

Having identified a localized binding hotspot within SPP1^41–90^, we next asked how this Ca^2+^-rich microenvironment could recruit phosphate ions from solution. For P_i_ recognition to occur, at least part of the Ca^2+^-loaded region must remain accessible to the surrounding solvent and thereby provide a possible interaction interface for incoming ions. To probe the existence of such an interface, we employed the recently developed HyperW-deconvolution method (Figure 3a), which enables residue resolution-level interrogation of IDPs by hyperpolarized NMR.^33,40–42^ In this method, the water in the buffer is replaced with ^1^H signal-amplified (hyperpolarized) water. To this end, we hyperpolarized water ex situ in a dedicated dynamic nuclear polarization (DNP) device and then transferred it to the NMR spectrometer, where it was mixed in situ with the SPP1^41–90^ solution. Proton exchange between HyperW and the peptide then leads to ^1^H signal enhancement of the latter (all details in the Experimental section in the Supporting Information). In post-processing, line shapes are then restored to their native width via a tailored deconvolution procedure (see Supporting Information Figure S3).

**Figure 3:**
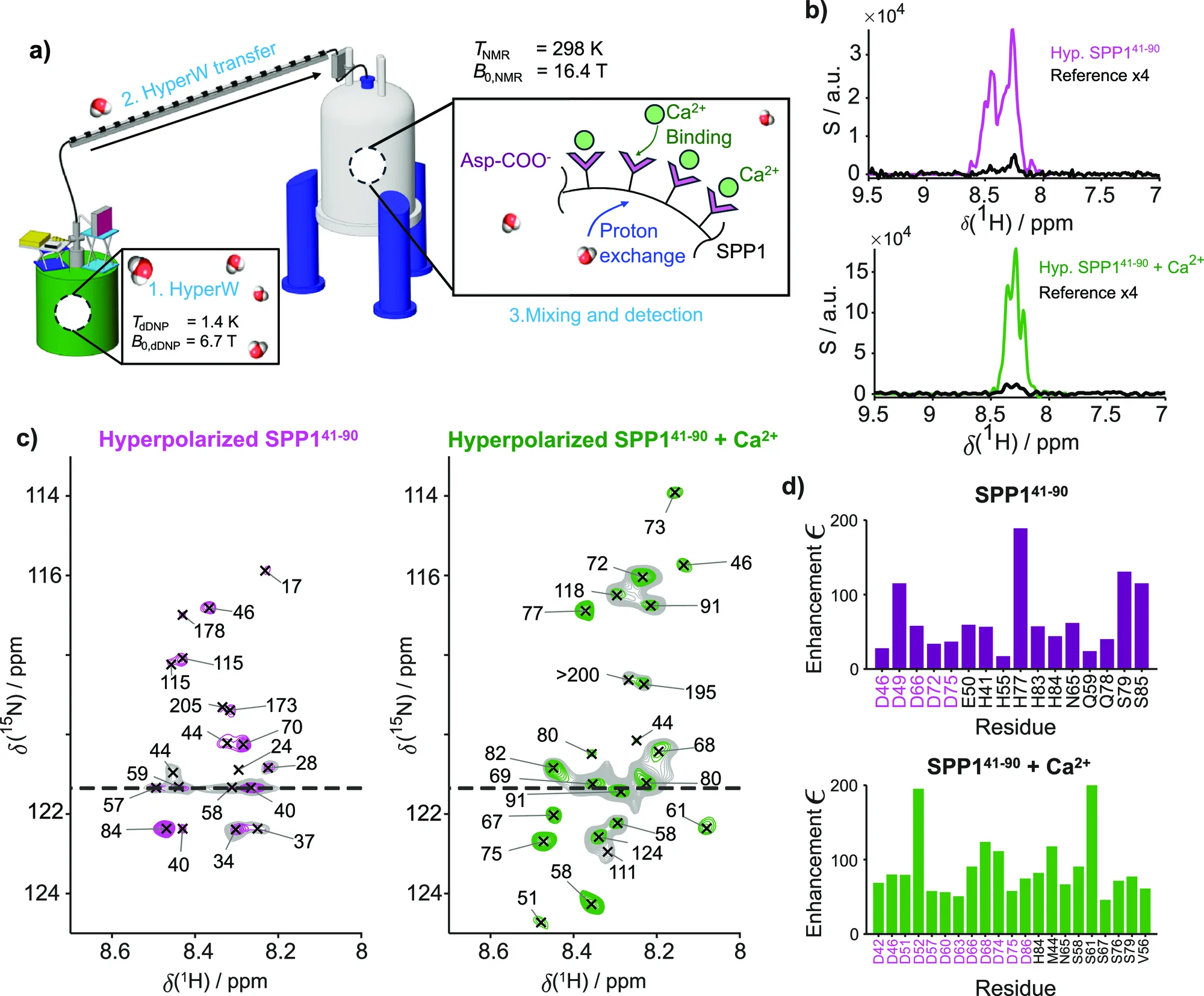
Conformational ensemble of Ca^2+^-associated SPP1^41–90^ by hyperpolarized NMR. **a)** Sketch of the experimental setup. 1. Hyperpolarized water (HyperW) with strongly enhanced ^1^H NMR signals is produced ex situ by dynamic nuclear polarization (DNP). 2. The HyperW is dissolved and transferred to an NMR spectrometer. 3. HyperW is mixed with SPP1^41–90^ waiting in the NMR tube in the presence or absence of Ca^2+^. HyperW-to-peptide proton exchange then transfers nuclear hyperpolarization, i.e., signal strength, from the solvent to the target molecule, which enables selective read-out of solvent-exposed residues. **b)** Effect of HyperW on the NMR signal strength. Top: Two-scan ^1^H NMR signal of SPP1^41–90^ in HyperW (magenta) and after decay of the hyperpolarization (black, same sample and experiment, four-fold enlarged). Clearly, the signal intensity has improved significantly in HyperW. Bottom: The same as in the top panel, but in the presence of Ca^2+^ (green). **c)** Hyperpolarized ^1^H–^15^N HMQC of SPP1^41–90^ in the absence (magenta/gray) and presence (green/gray) of Ca^2+^. For each cross-peak, the signal enhancement is indicated in the figure. Note that only a subset of residues is detected in HyperW (cf. Figure 2), albeit at much improved sensitivity, due to the dependence of signal strength on proton exchange efficiencies. Slow exchanging, shielded residues are not enhanced and remain below the detection threshold. In the presence of Ca^2+^, more peaks can be detected than in its absence. The dashed lines indicate where slices for panel b were taken. **d)** Residue-resolved signal enhancements in the absence (top) and presence (bottom) of Ca^2+^. D-type residues are highlighted. Note that Ca^2+^ exposure leads to the detection of a larger set of Asp residues (12 vs. 5). Thus, upon Ca^2+^ interaction, SPP1^41–90^ adopts a conformational ensemble in which the Asp residues receive more hyperpolarized protons, i.e., become preferably solvent-exposed. Gray contours are low-S/N resonances of the respective spectrum, drawn at a reduced threshold.

HyperW is particularly well suited to map solvent-interaction interfaces, as signal enhancement depends on solvent accessibility and proton exchange rates rather than directly reflecting structural populations. As a result, exposed residues with labile protons and fast exchange are preferentially detected, whereas shielded or slowly exchanging sites may often remain invisible.^29,40,43,44^

As shown in Figure 3b, HyperW dramatically increased signal intensities of solvent-exposed residues in both the free and Ca^2+^-bound states of SPP1^41–90^. Whereas NMR in normal water (*ceteris paribus*) detects only weak signals, replacement with HyperW readily yielded intense resonances. This sensitivity enhancement enabled the acquisition of 2D ^1^H^N^-^15^N spectra within only ∼25 s.

The hyperpolarized spectra are shown in Figure 3c (Boltzmann-polarized references in Supporting Information Figures S4–S6). Notably, only a subset of the resonances observed in the conventional NMR setting (cf. Figure 2) is detected via HyperW. This observation is consistent with previous reports^40,44^ indicating the presence of a partially compacted conformational state that shields large parts of the CBD from the solvent. The isolated CBD studied herein apparently retains this behavior, resulting in the detection of only a small set of residues (16 out of 50) that exhibit pronounced solvent accessibility.

This behavior changes drastically upon exposure to Ca^2+^. In the absence of Ca^2+^, only five Asp residues (D46, D49, D66, D72, and D75) were observed. Upon calcium exposure, this number increases to 12 Asp residues (D42, D46, D51, D52, D57, D60, D63, D66, D68, D74, D75, and D89; Figure 3d). Most of these newly detected residues are located within or directly adjacent to the calcium-interaction hotspot identified in Figure 2 (residues 48–55).

The pronounced increase in the number of detected Asp residues is consistent with Ca^2+^ binding involving a substantial reorganization of the conformational ensemble of SPP1^41–90^. Rather than merely associating with the acidic peptide, Ca^2+^ shifts the ensemble toward states in which a significantly larger fraction of Asp-rich regions becomes accessible to the solvent and, consequently, to HyperW-mediated polarization transfer. To confirm this data interpretation, we have also performed water-selective (WS) NOESY and ^15^N relaxation measurements (Supporting Information Figure S7). These data align with a picture in which the CBD exhibits greater conformational flexibility upon Ca^2+^ interaction, together with accelerated water interactions, supporting the interpretation that increased solvent exposure underlies the stronger signal enhancements.

However, it should be noted that changes in proton exchange due to counterion effects and changes in hydration dynamics alone (not entailing any conformational changes) cannot be entirely excluded. The observed increase in the HyperW-derived enhancement might involve a mixture of potentially unrelated conformational and hydration effects.

It should further be noted that the preferential detection of Asp residues does not mean that the residues between them are not solvent-exposed. Instead, our earlier work^42^ showed that amino acids with labile side-chain protons receive much stronger hyperpolarization from HyperW than aliphatic residues.

Taken together, we conclude that calcium binding generates a solvent-accessible Ca^2+^/Asp-rich interface that is positioned to recruit phosphate ions from solution during the earliest stages of CaP mineralization.

Consequently, we investigated whether these residues indeed participate in P_i_ recruiting. This represented a formidable methodological challenge as phosphate exposure rapidly triggered calcium phosphate formation and coprecipitation of SPP1^41–90^, thereby removing the relevant species from solution before conventional multidimensional NMR experiments could be completed.

HyperW, however, provided a straightforward solution for accessing the short-lived P_i_-encounter complexes. We conceived an experiment in which 0.5 M P_i_ (K_2_HPO_4_) was pre-dissolved in HyperW (already in the DNP magnet), thereby (upon dissolution) admixing it simultaneously with HyperW with the Ca^2+^-loaded SPP1^41–90^ complexes (yielding a Ca^2+^:P_i_ ratio of 1:2 in the final mixture^45,46^ ). This ratio led to the formation of brushite in the presence as well as in the absence of SPP1^41–90^ (see Supporting Information Figure S8).

Note that, in serum, total Ca^2+^ (∼2.5 mM) exceeds P_i_ (<1 mM), opposite to our conditions, which were chosen to efficiently precipitate the biomimetic model system under study on the time scale amenable for DDNP detection.

Thus, we recorded NMR spectra of the peptide right at the onset of coprecipitation with CaP (Figure 4a). In this way, hyperpolarized NMR detection captured its molecular state immediately after phosphate encounter and prior to CaP coprecipitation.

**Figure 4:**
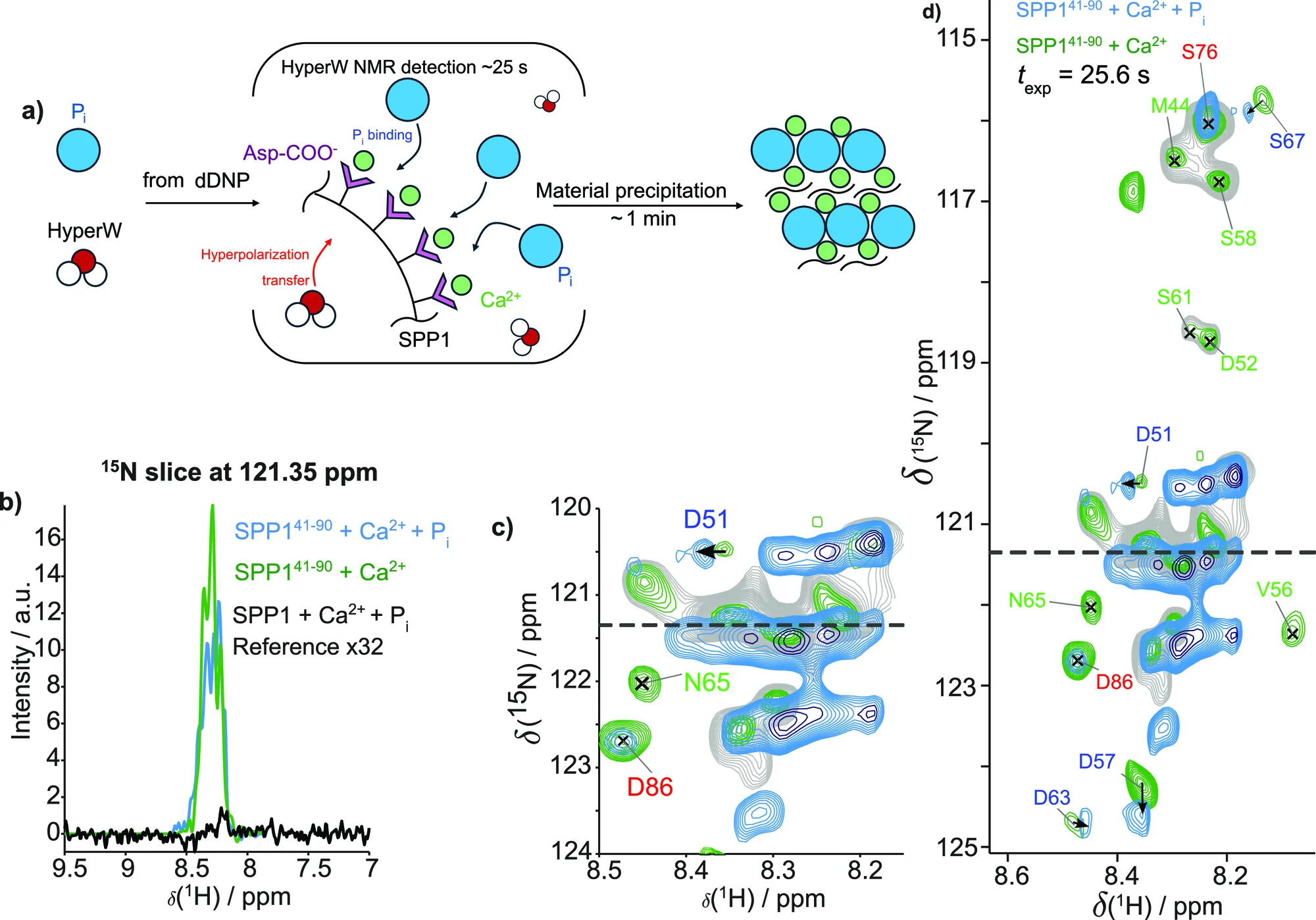
Transient hyperpolarized NMR upon P_i_ encounter. **a)** Sketch of the performed experiment and P_i_ interaction mode. P_i_ is dissolved in the water pellet used for DNP. Dissolution and mixing with Ca^2+^-coordinated SPP1^41–90^ then lead to P_i_ recruitment and CaP-SPP1^41–90^ composite formation. At the same time, boosted by HyperW, NMR detection within 25 s after phosphate encounter and before sample precipitation becomes possible. **b)** ^1^H spectra of SPP1^41–90^ in HyperW in the presence of Ca^2+^ (green/gray) and immediately after Pi encounter (black). P_i_ exposure leads to pronounced changes already in the 1D spectra. The thermal equilibrium, conventional reference spectrum is shown in black (32-fold amplified). The spectrum is very weak due to sample precipitation. **c, d)** Hyperpolarized ^1^H–^15^N HMQC spectra of Ca^2+^-bound SPP1^41–90^ in the absence (green) and immediately after encounter (blue/gray) with P_i_. The signal assignment before P_i_ interaction is indicated. The dashed lines show where the slice in panel b was taken. P_i_-encounter leads to a loss of resonances and significant chemical shift changes. Asp residues centered around amino acids 50–70 (i.e., including the Ca^2+^ interaction hotspot) show the strongest effects (shifts indicated by the arrows, blue labels D51, D57, D63; disappearing signals indicated by green labels M44, D52, V56, S58, S61, N65). In contrast, residues remote to this site (S76, S86, red labels) show little to no response. Gray contours are low-S/N resonances of the respective spectrum, drawn at a reduced threshold.

The signal enhancement obtained in these experiments is demonstrated via the ^1^H spectra displayed in Figure 4b. While strong resonances were readily observed in HyperW, the corresponding thermal-equilibrium reference spectrum, detected long after the completion of the HyperW experiment (ca. 5 min later), already exhibits only prohibitively weak signal intensity, even after 32-fold amplification, owing to the material loss through precipitation—an observation that highlights HyperW’s unique ability to probe transient states inaccessible to conventional NMR.

Figure 4c, d shows the corresponding hyperpolarized ^1^H–^15^N HMQC spectra (Boltzmann-polarized references in the Supporting Information Figures S4–S6). Relative to the Ca^2+^-bound state, phosphate addition leads to both strong chemical shift perturbations and the disappearance of resonances.

The P_i_-induced spectral changes fall into two distinct categories. Residues D51, D57, and D63 display pronounced chemical shift changes, consistent with fast-to-intermediate exchange contacts with incoming phosphate ions at the Ca^2+^-loaded interface. In contrast, residues M44, D52, V56, S58, S61, and N65 are broadened beyond detection, suggesting either slower exchange dynamics upon P_i_ association or the formation of larger, slowly tumbling encounter assemblies. Importantly, the residues that become newly observable upon Ca^2+^ exposure in HyperW (Figure 3) largely overlap with the region exhibiting the strongest Ca^2+^- and phosphate-induced perturbations (Figures 2 and 4), linking calcium ion association, conformational reorganization, and phosphate interaction to the same molecular hotspot.

Three further observations constrain the interpretation of the phosphate-induced perturbations, although full resonance assignment cannot be achieved on the short experimental time scale. First, a negative control (Figure S9) shows that P_i_ alone elicits no significant spectral response, establishing that phosphate interaction is strictly contingent on the prior Ca^2+^-organized state. Second, under the present 1:2 Ca^2+^:P_i_ conditions, bulk CaP formation proceeds on time scales compatible with the 25 s acquisition window.^45,46^ Third, the perturbations are spatially confined to the Ca^2+^-interaction hotspot (residues ∼50–70), whereas remote residues (S76, S86; cf. Figures 2f,3) remain unaffected. Taken together, these observations indicate that the detected species do not arise from bulk mineral precipitation passively depleting peptide from solution, but from a localized, Ca^2+^-templated phosphate recruitment event at the Asp interface. Finally, it should be noted that our data cannot distinguish initial phosphate coordination from subsequent phosphate-mediated bridging of adjacent Ca^2+^-coated interfaces. Nonetheless, the broadening of several residues (M44, D52, V56, S58, S61) is most consistently attributed to local assembly of phosphate-bridged, Ca^2+^-loaded peptide segments, placing this event upstream of peptide-guided CaP formation.

## Conclusions

Together, our observations support a sequential model in which Ca^2+^ binding first reorganizes the conformational ensemble of SPP1’s CBD, exposing a set of counterion-coated acidic Asp residues that subsequently serve as an anion-capturing interface. However, the reported phenomena are not exclusive to CaP precipitation. SPP1 is known to also be involved, e.g., in calcium carbonate precipitation.^47^ Hence, similar observations for other oxyanions are possible.

Methodologically, we show that residue-resolved hyperpolarized NMR can provide residue-resolved access to transient peptide–ion assemblies immediately preceding mineral precipitation, thereby opening new opportunities to investigate the molecular mechanisms underlying peptide-guided biomineralization and to unravel its early stages.

## Supplementary Material



## Notes

Materials and Methods section, calculations of dDNP enhancements and CSP, sample preparation for SEM, sample preparation and spectra for FTIR, Ca^2+^ titration spectra, DOSY curves, details on FID deconvolution and exemplary WS-NOESY spectra, *R*_2_ rates for selected residues (PDF)
