# Supplementary material for: Following Transient Conformational Adaptations during Peptide-Guided Calcium Phosphate Biomineralization with Hyperpolarized NMR
Source: JACS Au. 2026 Sep 16;6(9):5401–9. doi: 10.1021/jacsau.6c01076 (PMC13625547; doi:10.1021/jacsau.6c01076)
Supplement: Supplementary file 1 [file au6c01076_si_001.pdf]

Following transient conformational adaptations during peptide-guided calcium phosphate biomineralization with hyperpolarized NMR

*Fanny Kormout[1,2,+], Ertan Turhan[1,+], Mehdi Soussi-Therond[1], Milan Zachrdla[1], Dennis Kurzbach[1,2]**

[1]University of Vienna, Faculty of Chemistry, Institute of Biological Chemistry, Währinger Str. 38, 1090 Vienna, Austria.

[2]Doctoral School of Chemistry (DoSChem), University of Vienna, Faculty of Chemistry, Währinger Str. 38, 1090 Vienna, Austria

**corresponding author: dennis.kurzbach@univie.ac.at*

+equal contributions

Supporting Information

Experimental Section

Sample Production

The calcium-binding domain of human SPP1 comprising residues 41–90 (SPP1$^{41–90}$; according to https://www.uniprot.org/uniprotkb/Q3LGB0/entry ; note that different entries exist with different lengths) was expressed as a His-tagged SUMO fusion protein containing a TEV protease cleavage site using a pET-21a(+) expression vector in E. coli Rosetta2 cells. For isotopic labeling, cells were grown in M9 minimal medium supplemented with $^{15}NH_4Cl$ (1 g/L) as the sole nitrogen source. Protein expression was induced with isopropyl β-D-1-thiogalactopyranoside (IPTG) at an optical density of A_{600} = 0.7, and cultures were incubated overnight at 30 °C.

Cells were harvested and lysed in 25 mM Tris-HCl, 100 mM NaCl, and 2 mM β-mercaptoethanol (pH 8.0). The clarified lysate was purified by Ni^{2+}-affinity chromatography, and fractions containing the His-SUMO-SPP1$^{41–90}$ fusion construct were identified by LC-MS and pooled. Removal of the His-SUMO tag was achieved by overnight digestion with TEV protease. Complete cleavage was verified by LC-MS analysis. The sample was subsequently subjected to a second Ni^{2+}-affinity chromatography step to remove the cleaved tag, uncleaved fusion protein, and TEV protease. Fractions containing SPP1$^{41–90}$ were identified by LC-MS (Fig. S10), pooled, and lyophilized prior to NMR experiments.

For NMR and dDNP experiments, SPP1$^{41-90}$ (sequence (G) HDHMD DMDDE DDDDH VDSQD SIDSN DSDDV DDTDD SHQSD ESHHS DESDE, MW = 5774 Da) was dissolved at a concentration of 2 mg mL^{-1} (0.346 mM) in 50 mM HEPES buffer (pH 6.5). Samples were prepared in Shigemi NMR tubes using a total sample volume of 350 µL containing 10% (v/v) D_2O for spectrometer lock. Ca^{2+} loading was achieved by stepwise addition of 5 µL aliquots of a concentrated $CaCl_2$ stock solution while maintaining a constant pH of 6.5. Final $CaCl_2$ concentrations corresponded to peptide:Ca^{2+} ratios between 2 and 60 equivalents (0.70–21.0 mM $CaCl_2$). After each addition, samples were mixed thoroughly and allowed to equilibrate prior to NMR measurements. Control samples were prepared identically in the absence of $CaCl_2$.

To produce hyperpolarized water, 200 µL aliquots of a mixture of 85% dd-H_2O, 15% glycerol-d_8 and 15 mM TEMPOL were subjected to DNP.

For phosphate-encounter experiments, 0.5 M K_2HPO_4 were dissolved directly in the water/glycerol mixture prior to dissolution and was therefore introduced simultaneously with the hyperpolarization reservoir into the Ca^{2+}-loaded SPP1$^{41–90}$ solution. This resulted in a final P_i concentration of 12.8 mM upon detection.

NMR Spectroscopy

HSQC two-dimensional ^1H–^{15}N correlation spectra were recorded on a Bruker Avance Neo 600 MHz spectrometer operating at a ^1H Larmor frequency of 600.18 MHz and equipped with a triple-resonance TCI cryogenic probe (H/F-C/N-D). All experiments were performed at 298 K. All residue-specific backbone spectra and calcium-induced chemical shift perturbations were obtained from sensitivity-enhanced gradient-selected HSQC spectra using the Bruker pulse sequence *hsqcetf3gpsi*. Spectra were recorded with 2048 complex points in the direct (^1H) dimension and 256 increments in the indirect (^{15}N) dimension using spectral widths of 9615 Hz (16.0 ppm, centered around 4,7 ppm) and 2129 Hz (35.0 ppm, centered around 120 ppm), respectively. Four scans were acquired per increment with a recycle delay of 1.0 s and two dummy scans, resulting in a total acquisition time of approximately 18 min per spectrum. Data were processed in TopSpin 5.0 (Bruker BioSpin) and MATLAB. The indirect dimension was reconstructed using States-TPPI processing and extended by forward linear prediction (32 complex coefficients, 128 output points) prior to Fourier transformation. Data were zero-filled to 256 points and apodized with a 60° shifted sine-bell window function. Spectra were phased and baseline corrected using a fifth-order polynomial function.

Chemical shift perturbations (CSPs) upon Ca^{2+} addition were calculated according to:

$$\Delta\delta = \sqrt{(\Delta\delta_H)^2 + (0.1\,\Delta\delta_N)^2}$$

where $\Delta\delta_H$and $\Delta\delta_N$ denote the observed ^1H and ^{15}N chemical-shift differences, respectively. Resonance assignment was achieved through BMRB entry 19999.

The resulting titration profiles were fitted using an empirical Hill equation:

$$\mathrm{CSP} = a + (b - a)\cdot[\mathrm{Ca}^{2+}]^{nH} / (\mathrm{K_d}^{nH} + [\mathrm{Ca}^{2+}]^{nH})$$

where *a* and *b* represent the lower and upper CSP plateaus, respectively, K_d is the apparent midpoint of the transition (expressed in equivalents of Ca^{2+}), and n_H is the apparent Hill coefficient. Nonlinear least-squares fitting was performed in MATLAB.

The Hill model was employed as a phenomenological description of the Ca^{2+}-dependent response and was not intended to imply a specific binding stoichiometry. For highly acidic intrinsically disordered proteins such as SPP1, apparent Hill coefficients greater than unity may arise from progressive charge neutralization, multivalent Ca^{2+} association, or collective conformational changes within the protein ensemble. Consequently, the fitted Hill coefficients should be interpreted as measures of the cooperativity and steepness of the observed transition rather than the number of Ca^{2+} ions bound per protein molecule.

The fitted parameters yielded apparent Hill coefficients of 4.08, 4.12, and 9.9 for residues E90, S61, and D52, respectively.

Diffusion coefficients were determined by diffusion-ordered NMR spectroscopy (DOSY) on a Bruker Avance Neo 600 MHz spectrometer at 298 K. Experiments were performed using a pulsed-

field-gradient stimulated-echo DOSY sequence with a linear array of 16 gradient strengths. The diffusion delay (Δ) was set to 200 ms and the gradient pulse duration (δ) to 5 ms. Spectra were recorded with 32 scans per gradient increment and processed using the Bruker DOSY analysis package. Diffusion coefficients were obtained by fitting the signal attenuation as a function of gradient strength to the Stejskal–Tanner equation as implemented in GNAT.[1]

^{15}N R_2 rates were measured at 298 K on a Bruker Avance Neo spectrometer operating at 600.18 MHz ^{1}H Larmor frequency (14.1 T) and equipped with a 5 mm TCI cryogenic probe, using the sensitivity-enhanced, gradient-selected ^{1}H–^{15}N CPMG experiment (hsqct2etf3gpsi3d) with echo–antiecho quadrature detection in the indirect dimension and water flip-back solvent suppression. Spectra were recorded with spectral widths of 16.0 ppm (9615 Hz, ^{1}H) and 35.0 ppm (2129 Hz, ^{15}N), carriers at 4.70 ppm (^{1}H, on the water resonance) and 117.0 ppm (^{15}N), and 1024 × 64 complex points, giving acquisition times of 106.5 ms (t_2) and 30.1 ms (t_1). INEPT transfers were set with $^{1}J(NH)$ = 90 Hz. Thirty-two transients were averaged per increment with a 1.0 s recycle delay, and ^{15}N was decoupled during acquisition using GARP (1.47 kHz field).

The CPMG block consisted of loop units of 16 ^{15}N refocusing pulses separated by 2 × 450 μs delays, giving a constant echo spacing of 1.06 ms (ν = 472 Hz) and 16.96 ms per unit; a ^{1}H 180° pulse was applied once per unit to suppress cross-correlated ^{1}H–^{15}N dipole/CSA relaxation. Loop counters of 1, 4, 8, 16, 20, 24, 28 and 38 gave relaxation delays of 17.0, 67.8, 135.7, 271.4, 339.2, 407.0, 474.9 and 644.5 ms, with the 67.8 and 407.0 ms points recorded in triplicate.

Data were processed in TopSpin 4.1.4, apodized with a squared cosine-bell window in both dimensions; the ^{15}N dimension was extended by forward linear prediction (64 coefficients) before zero-filling, giving final matrices of 2048 × 512 real points. Peak intensities were taken as the maximum within ±0.025 ppm (^{1}H) and ±0.25 ppm (^{15}N) of each assigned resonance, with assignments transferred from a reference ^{1}H–^{15}N HSQC and verified manually in every plane; overlapped or ambiguous resonances were excluded. Replicate delays were averaged and their standard deviation taken as the experimental uncertainty. Rates were obtained by nonlinear least-squares fitting of $I(t) = I_0 \cdot \exp(-R_2 \cdot t)$ in MATLAB 2025a, with uncertainties given by the standard error of the fitted R_2. ΔR_2 was calculated as $R_2(+Ca^{2+}) - R_2(-Ca^{2+})$ with errors propagated in quadrature.

WS-NOESY were measured as described in reference [2]. Water magnetization was manipulated by 80 ms Gaussian selective pulses centered on the water resonance (4.70 ppm) and allowed to transfer to the amide protons during a mixing period of 0, 250 or 500 ms before detection; the amide signal recovered in this way reports on both chemical exchange and cross-relaxation with water, and no attempt was made here to separate the two contributions as proton exchange largely dominates the polarization transfer. Detection used a fast HSQC with water flip-back and 3-9-19 WATERGATE solvent suppression, with spectral widths of 16.0 ppm (9615 Hz, ^{1}H) and 18.0 ppm (1095 Hz, ^{15}N), carriers at 4.70 and 120.0 ppm, and 1024 × 64 complex points (acquisition times 106.5 and 58.5 ms), States-TPPI quadrature detection in the indirect dimension, 32 transients per increment, a recycle delay of 2.0 s, and GARP ^{15}N decoupling (1.47 kHz) during acquisition. Each two-dimensional spectrum required approximately 3 h. The full series was recorded on both the apo and Ca^{2+}-loaded samples at 288 and 308 K (15 and 35 °C), with the sample allowed to equilibrate for 10 min after each temperature change.

Spectra were processed identically with a squared cosine-bell window and zero-filling in both dimensions. For each residue *r*, condition *c* (apo or Ca^{2+}-loaded) and temperature *T*, let $I(\tau_{mix})$ denote the peak intensity in the water-selective spectrum recorded with mixing time τ_{mix}. The build-up shape was quantified by the normalized intensity difference between the two non-zero mixing times,

$$\Delta WS_{500\text{-}250} = [\, I(\tau_1) - I(\tau_2) \,] / [\, I(\tau_1) + I(\tau_2) \,],\ \tau_1 = 500\ \text{ms},\ \tau_2 = 250\ \text{ms}$$

which is dimensionless, bounded on [−1, 1], and independent of any factor that scales both spectra equally — protein concentration, receiver gain, probe tuning, or the overall efficiency of the selective transfer.

FTIR

For FTIR measurements, a peptide solution was prepared in 0.05 M HEPES pH 6.5 to a final concentration of 0.1 mM SPP1, to which 8.6 mM CaCl2 was added and incubated for 30 min at room temperature before the addition of 12.9 mM K_2HPO_4 and another 30 min incubation period at room temperature. Then the suspension was centrifuged at 16 873 rcf for 5 min to form a pellet, which was washed with 1 mL of water. After another washing step in the same manner, the pellet was dried in the oven at 80°C for 1h. The spectra of the dried samples were recorded on a Bruker Tensor 37 FTIR spectrometer using a platinum ATR unit with a diamond crystal. 64 scans with a resolution of 2 cm^{-1} were averaged to obtain one spectrum.

Hyperpolarized NMR

Hyperpolarized water was generated by dissolution dynamic nuclear polarization (dDNP). 200 µL of a 15 mM TEMPOL solution in H2O/glycerol-d8 (85:15, v/v; optionally supplemented with 0.5 M K_2HPO_4) was polarized at 1.4 K and 6.7 T for 1 h using continuous-wave microwave irradiation at 188 GHz. Samples were freshly prepared prior to each experiment to avoid ripening.[3] DNP was performed using a home-built dDNP polarizer equipped with a Cryogenic Ltd. magnet–cryostat system and a VDI microwave source delivering approximately 50 mW microwave power at the sample.[4] Sample injection into the NMR tube waiting in the NMR spectrometer was achieved with the prototype injection system described in [5].

Following polarization, samples were dissolved with 5 mL D_2O under 15 bar pressure and transferred to the NMR spectrometer using pressurized helium gas. The HyperW was subsequently injected into the target solution within approximately 2 s using the HySSS.v2 prototype.[5-6] Protein samples contained $SPP1^{41\text{–}90}$ at 2 mg/mL prior to mixing with hyperpolarized solvent, optionally with 60 eq. $CaCl_2$. Upon injection of 300 µL hyperpolarized water into 150 µL protein solution, the final sample volume was 450 µL, corresponding to a peptide concentration of 0.67 mg mL^{-1}. The final H_2O concentration was 4%.

For detection, ^1H–^{15}N SOFAST HMQC[7] correlation spectra (*sfhmqcf3gpph*) were recorded on a Bruker Avance III HD 700 MHz spectrometer operating at a ^1H Larmor frequency of 700.40 MHz and equipped with a QCI cryogenic probe (H/F-C/N-D). All experiments were performed at 298 K. Spectra were recorded with 1024 complex points in the direct (^1H) dimension and 256 increments

in the indirect (^{15}N) dimension using spectral widths of 11160.7 Hz (15.9 ppm centered around 4.7 ppm) and 2483.9 Hz (35.0 ppm, centered around 120 ppm), respectively. Two scans were acquired per increment without dummy scans. Hyperpolarized datasets were recorded immediately following injection of the hyperpolarized solvent into the NMR sample using an automated TTL trigger that launched the pulse sequence upon completion of the mixing process. Excitation was achieved using a PC9 pulse with a duration of 2.4 ms and inversion using an RSNOB pulse with a duration of 0.8 ms, both centered around 8.3 ppm.

For data processing, the deconvolution routine described in reference [5, 8] was employed. In brief, the complex 2D FID was apodized with a 60°- shifted squared sine bell, Fourier-transformed, and baseline-corrected along the F2 dimension. The resulting interferograms were then divided by the decay function of the hyperpolarized water along F1 (identified through the deep learning approach outlined in [8]), truncated at a t_1 evolution time of 9 ms, followed by linear prediction, to 256 complex points (using 8 complex coefficients). followed by apodization with a q-sine (q=3) function and baseline correction using spline interpolation. Home-written scripts in MATLAB were used for this purpose.

Enhancements were calculated as

$$\varepsilon = S_{\mathrm{Hyp}}/S_{\mathrm{Ref}} \cdot \mathrm{NS}_{\mathrm{Ref}}/\mathrm{NS}_{\mathrm{Hyp}}$$

Using the same sample for hyperpolarized and reference data. *S* is the signal amplitude and NS the number of scans.

Note that the enhancements reported here are referenced to the thermal-equilibrium signal recorded under unchanged buffer conditions, so that the high degree of solvent deuteration (96%) cancels from the ratio. Deuteration does, however, reduce the absolute ^{1}HN amide signal: relative to a sample at 10% deuteration, the thermal-equilibrium resonance would be 22.5-fold more intense. The net sensitivity gain of HyperW over a conventional protonated experiment is thus smaller than the enhancement factor by approximately this amount. For the purpose at hand, the relevant information is contained in the relative changes in enhancement across HyperW experiments, for which deuteration is a common factor.

Scanning Electron Microscopy

For SEM measurements, a peptide solution was prepared in 0.05 M HEPES pH 6.5 to a final concentration of 0.1 mM $SPP1^{41-90}$, to which 8.6 mM $CaCl_2$ was added and incubated for 30 min at room temperature before the addition of 12.9 mM K_2HPO_4 and another 30 min incubation period at room temperature. Then the suspension was centrifuged at 16 873 rcf for 5 min to form a pellet, which was washed with 1mL of water. After another washing step in the same manner, the pellet was resuspended in 150 µL of ddH_2O and 30 µL each were applied to cover slips previously washed with 70% EtOH and dried. The suspension was spread onto the coverslip as a thin layer and then dried in the oven at 80°C for 1h. A layer of gold was added to all samples by sputter coating under high vacuum (Bal-Tec SCD 005), and SEM imaging was performed with a Zeiss SEM supra 55 VP at 5 kV.

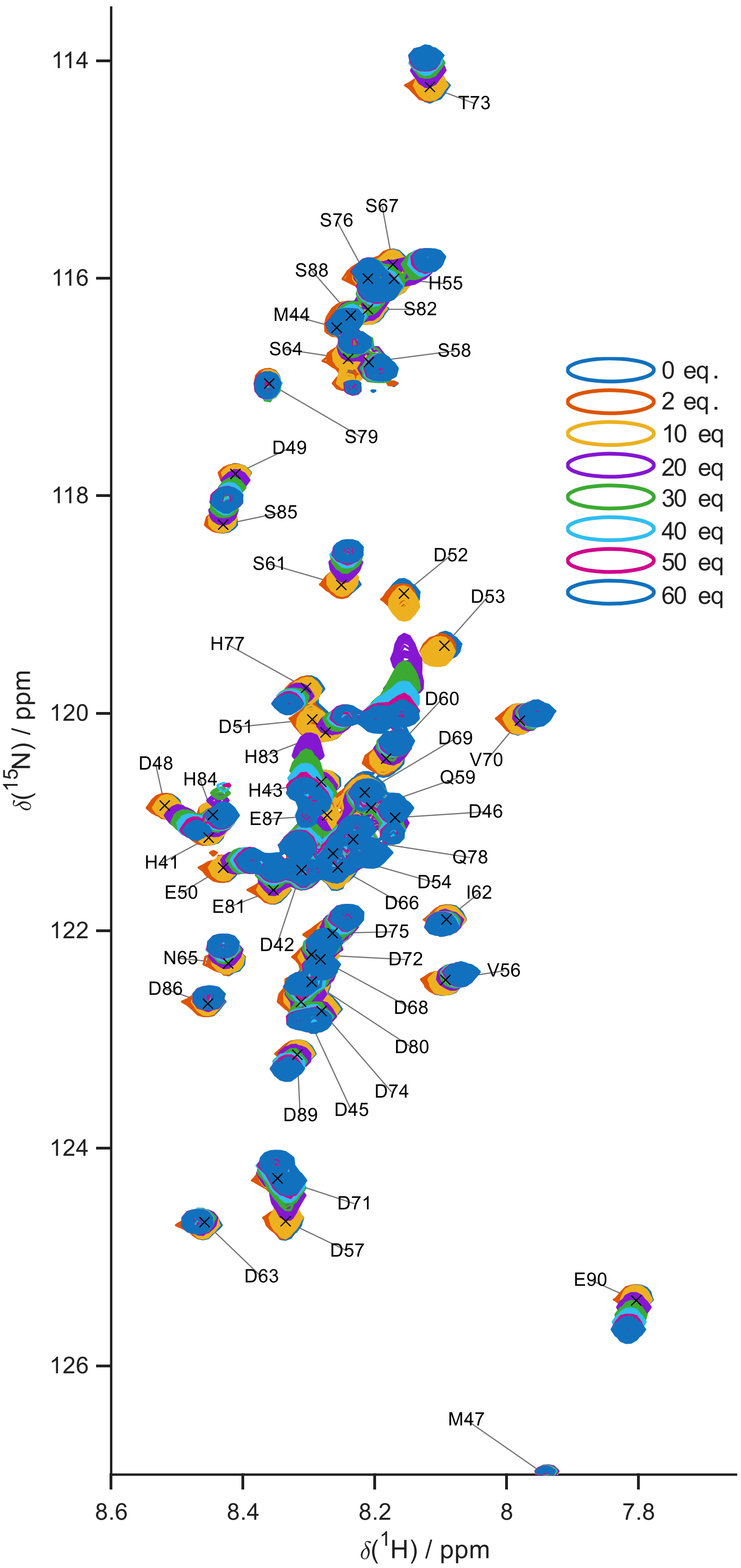

Figure S1. Overlay of all Ca^{2+} titration spectra to follow the cross peaks.

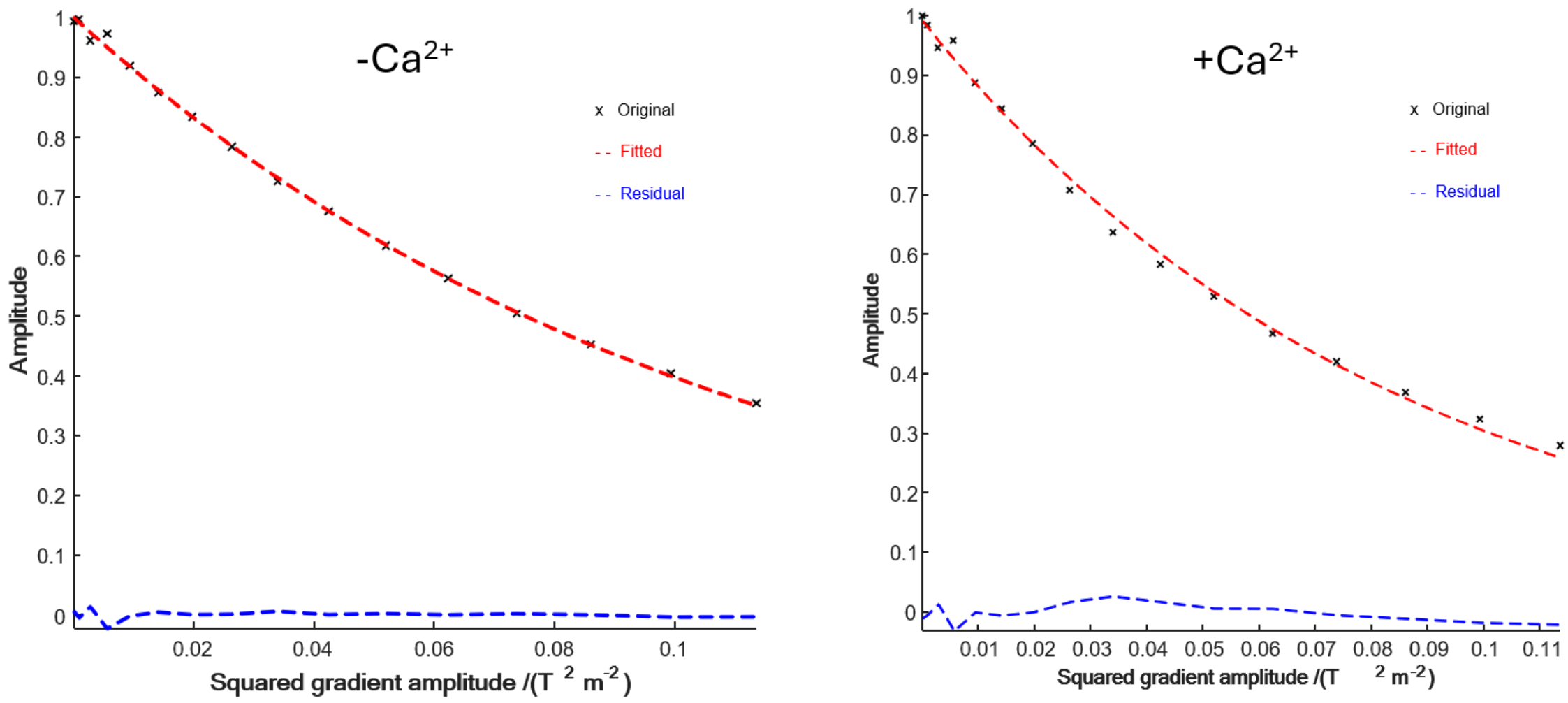

Figure S2. DOSY curves of SPP1[41-90] with and without 60 eq. of Ca^{2+}. The data were fitted to diffusion coefficients of 2.21+/-0.08 and 1.90 +/- 0.26 10^{-10} m^2/s. No significant aggregation is detected by DOSY.

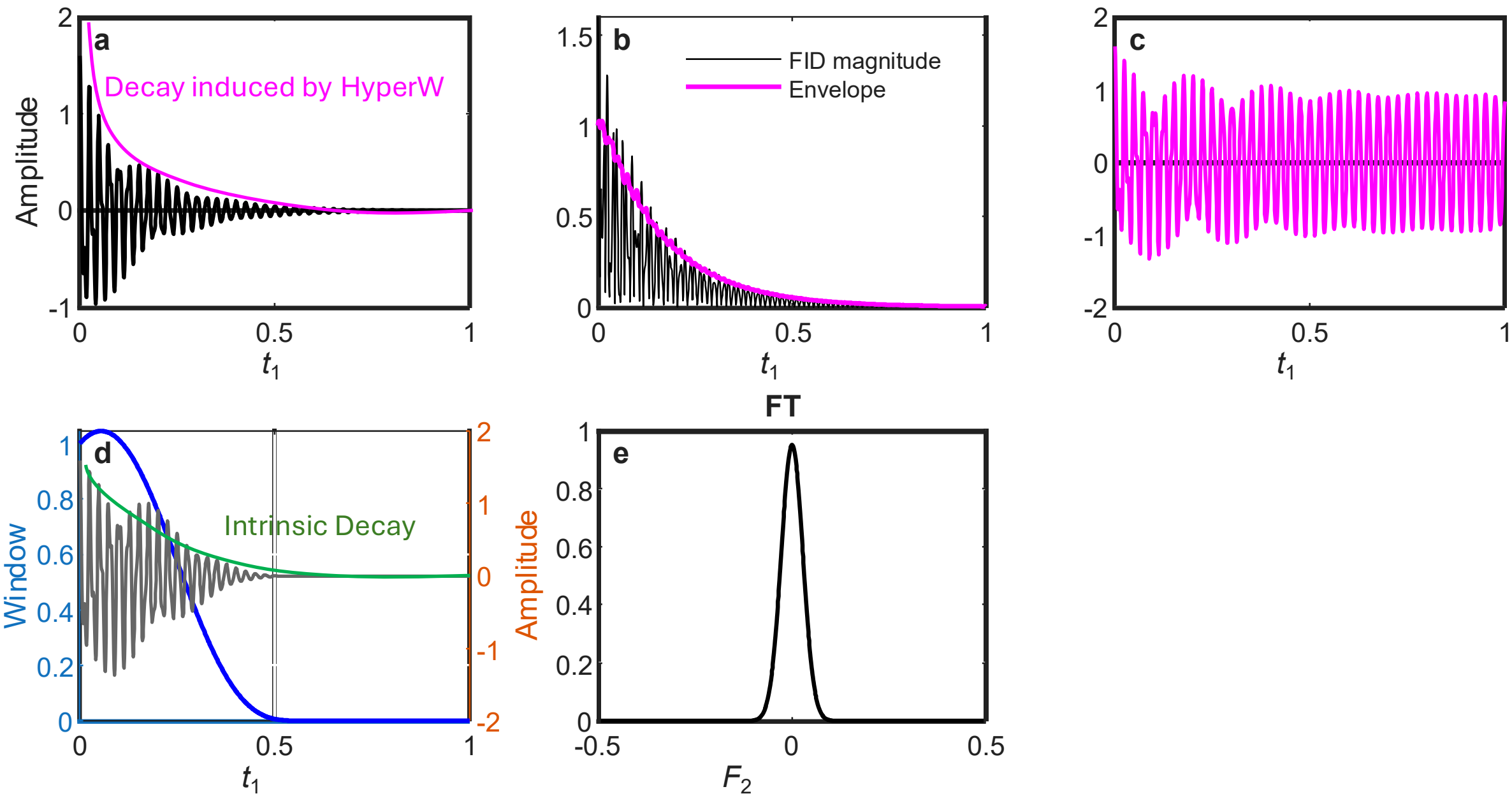

Figure S3. Schematic illustration of the FID deconvolution procedure. A synthetic free induction decay (FID) is used to demonstrate the processing workflow. (a) Raw time-domain FID exhibiting signal decay along t_1, which is accelerated by the decay of the hyperpolarized water. (b) Estimation of the signal envelope from the FID magnitude as described in detail in reference [8]. (c) Deconvolution by division of the FID with the normalized envelope, thereby removing the decay component induced by HyperW. (d) Application of a q-sine apodization function (blue) and removal of noise at long t_1, introduced by the deconvolution, yielding the apodized time-domain signal (black). (e) The Fourier transform of the processed FID yields the final spectrum. The figure is intended solely to illustrate the sequence of processing steps and does not represent experimental data.

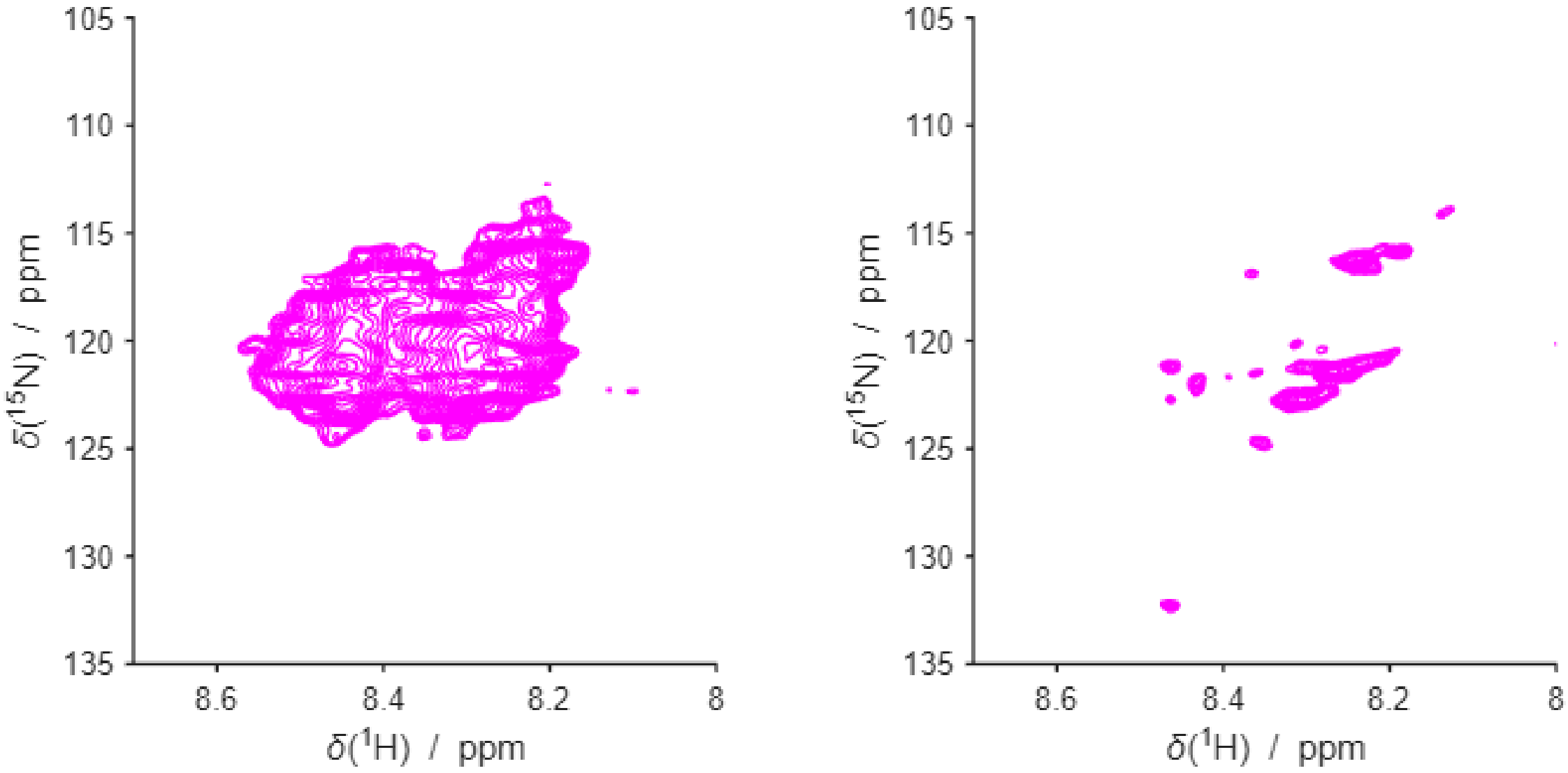

Figure S4. Spectrum of SPP1$^{41-90}$ in HyperW without deconvolution and the thermal reference recorded on the same sample with the same pulse sequence. The bottom contour level in both spectra is the same above the noise floor, allowing direct comparison. NS = 2 and the degree of deuteration was 96% in both cases.

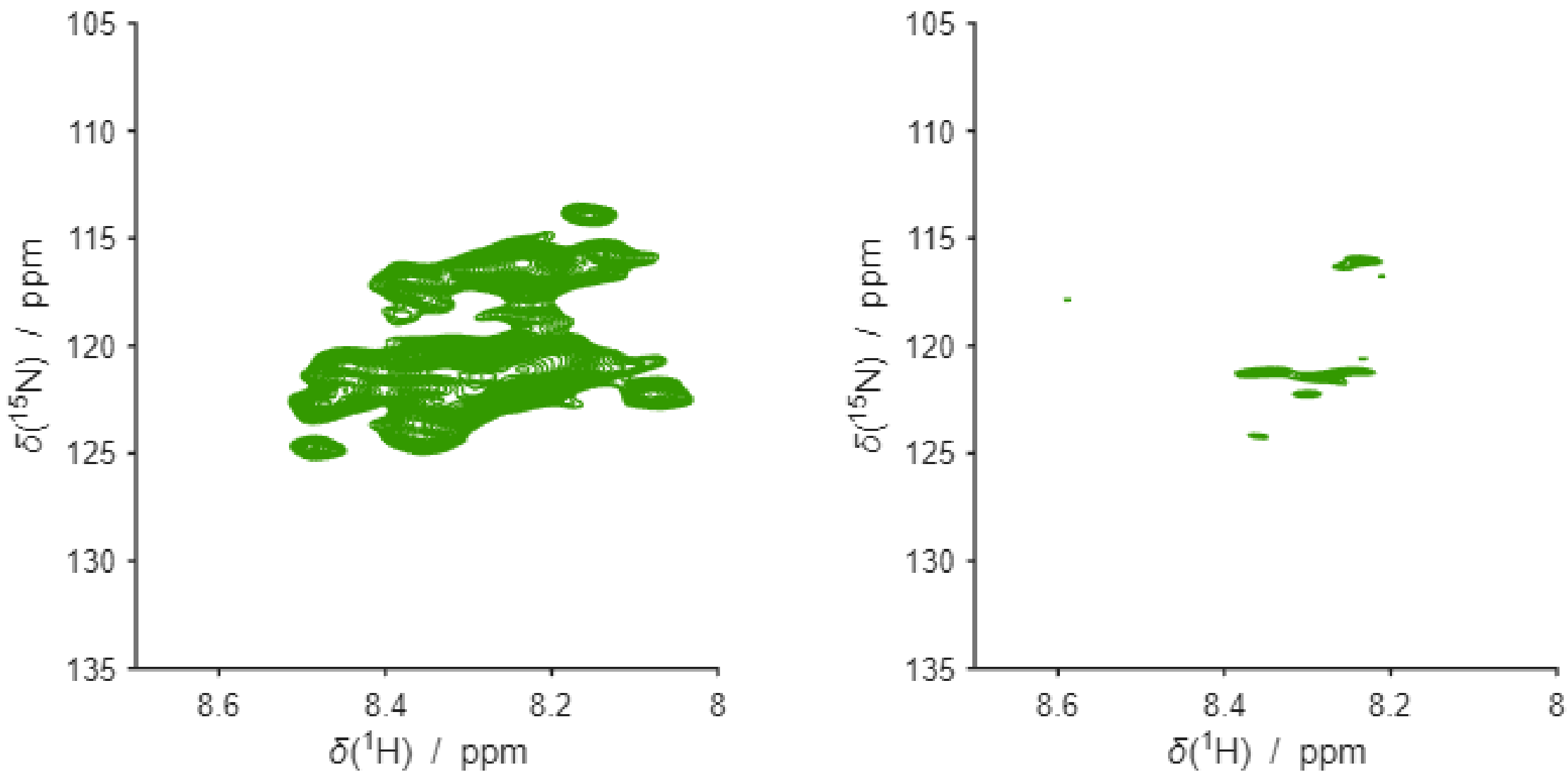

Figure S5. Same as Fig. S4, but in the presence of 60 eq. Ca^{2+}.

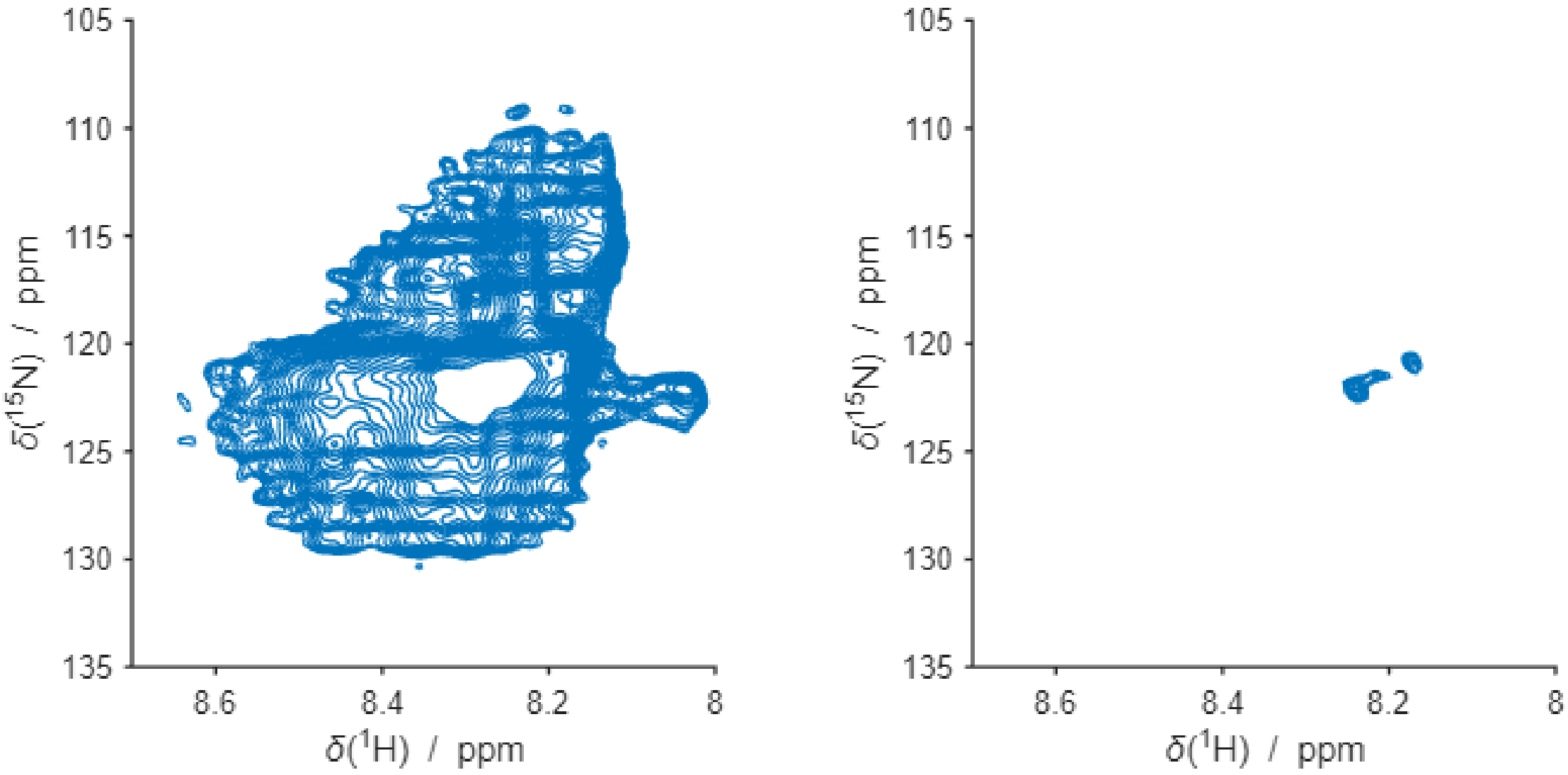

Figure S6. Same as Fig. S4, but after P_i encounter.

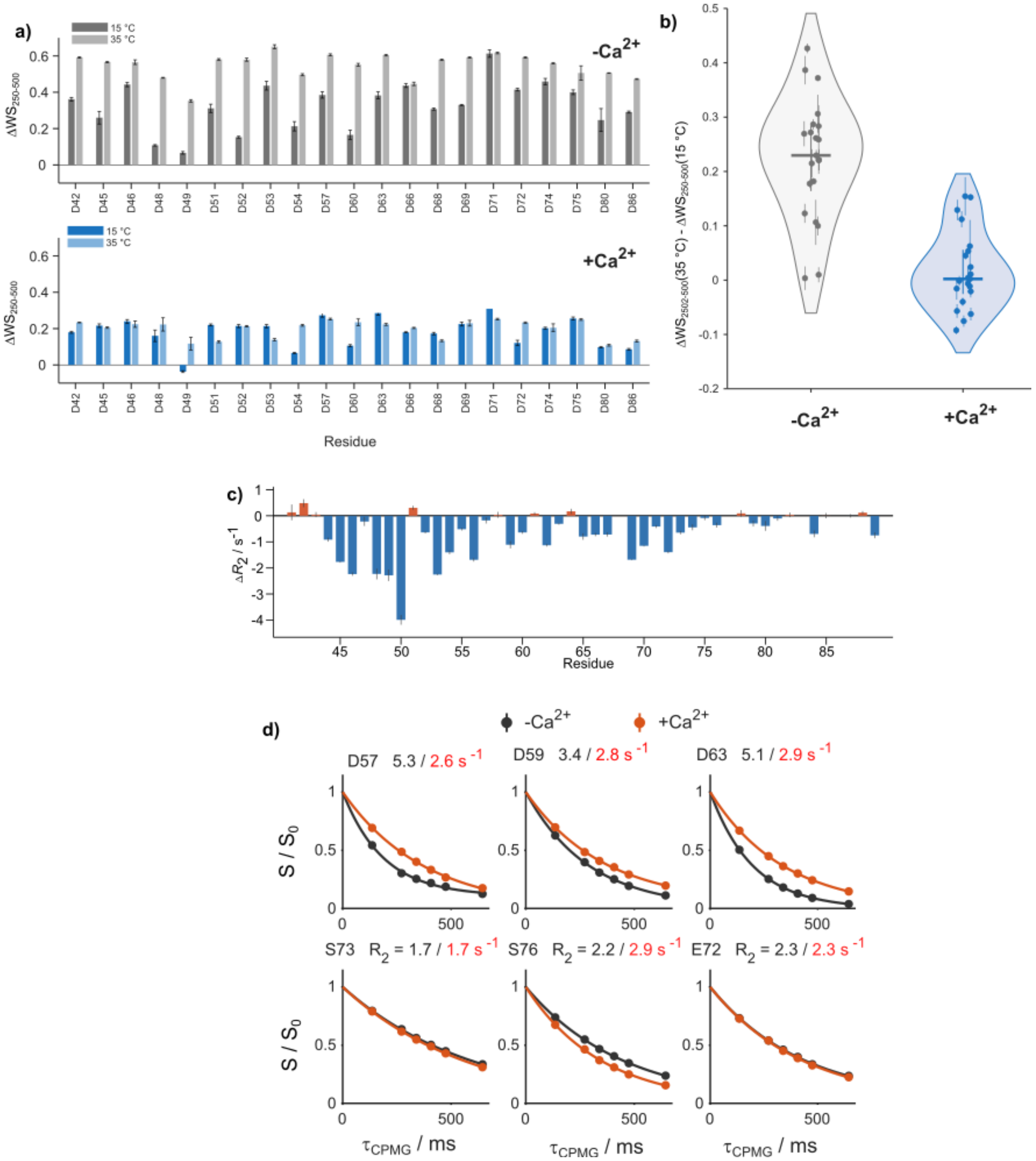

Figure S7. a) WS-NOESY data of the Asp-residues of SPP1$^{41\text{-}90}$. Normalized intensity difference between τ_{mix} = 250 and 500 ms $\Delta WS_{500\text{-}250}$ measured at 15 and 35 °C as well as in the absence (top, grey) and presence (bottom, blue) of Ca^{2+}. Upon addition of Ca^{2+}, a notably different temperature dependence is observed. b) Violin plot of the temperature effect on the slope of the WS-NOESY build-up expressed as $\Delta WS_{500\text{-}250}$ (35 °C) - $\Delta WS_{500\text{-}250}$ (15 °C) in the absence (grey) and presence (blue) of Ca^{2+}. The temperature dependence of the buildup differs markedly for almost all Asp residues. Thus, a mere increase in exchange rates upon Ca^{2+} exposure is unlikely. Assuming proton exchange as the major contribution to the polarization transfer and that $k_{ex} \propto \exp(-E_A/k_BT)$, a similar temperature response would be expected in such a case. c) Differential ^{15}N relaxation rates along the entire peptide strand defined as $\Delta R_2 = R_2(+Ca^{2+}) - R_2(-Ca^{2+})$. Along the primary sequence, and particularly within the Ca^{2+} binding sites, the values are negative, indicating greater conformational flexibility upon exposure to Ca^{2+}. This supports the interpretation of the DDNP data with a model based on increased solvent exposure, not mere changes in proton exchange rates. **d**) Exemplary decay curves of ^{15}N-R_2 relaxation rate measurements. Within the Asp-rich Ca^{2+}-binding site (top row) R_2 rates become shorter upon

addition of Ca^{2+} leading to a negative $\Delta R_2 = R_2(+Ca^{2+}) - R_2(-Ca^{2+})$. Other, not-involved residues show little to no change (bottom row).

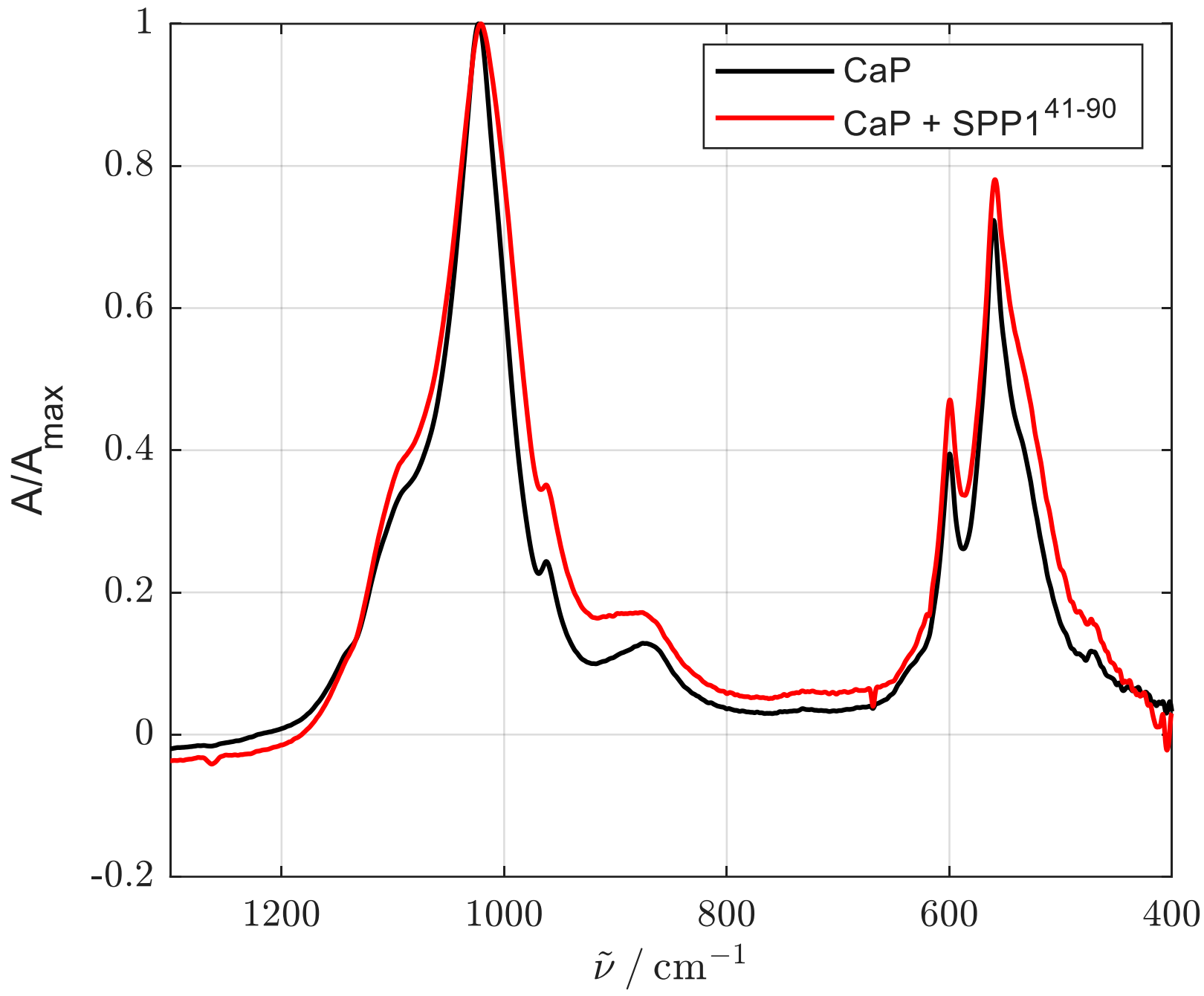

Figure S8. FTIR of the precipitated CaP. The signature is characteristic of brushite in the absence (black) and presence of SPP1$^{41\text{-}90}$ (red).[9]

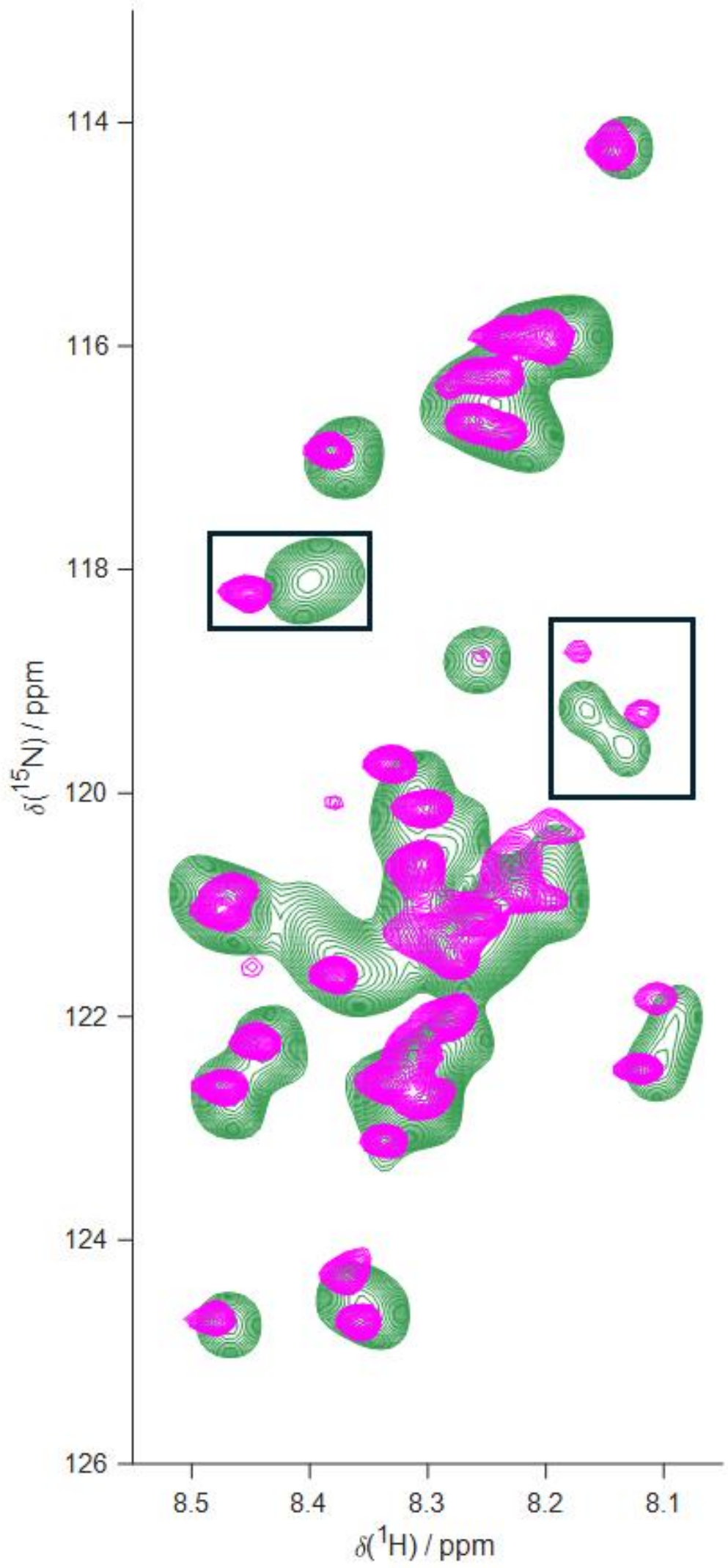

Figure S9. Negative control of injecting P_i together with HyperW into an SPP1$^{41\text{-}90}$ solution, but in the absence of any $CaCl_2$. The spectrum (green) matches that of neat SPP1$^{41\text{-}90}$ (magenta) with the exception of 3 resonances (black squares, likely due to the presence of P_i), showing that calcium-incubation is necessary to lead to the chemical shift perturbations upon phosphate encounter described in the main text. Note that the magenta spectrum was recorded in 90% H_2O/10% D_2O with 4 scans, whereas the magenta reference in Fig. S4 was recorded with 2 scans and in 4% H_2O/96% D_2O. Thus, different intensities are obtained.

We emphasize that the Ca^{2+} dependence does not imply selectivity for phosphate over other oxyanions, but only a sequential process. SPP1 also regulates calcium carbonate mineralization in the avian eggshell[10] through the same class of acidic motifs, and the Ca^{2+}-loaded Asp interface is expected to recruit oxyanions generically. The sequential dependence demonstrated here — Ca^{2+} binding preceding and enabling P_i recruitment — is a statement about the order of the two events, not about anion discrimination.

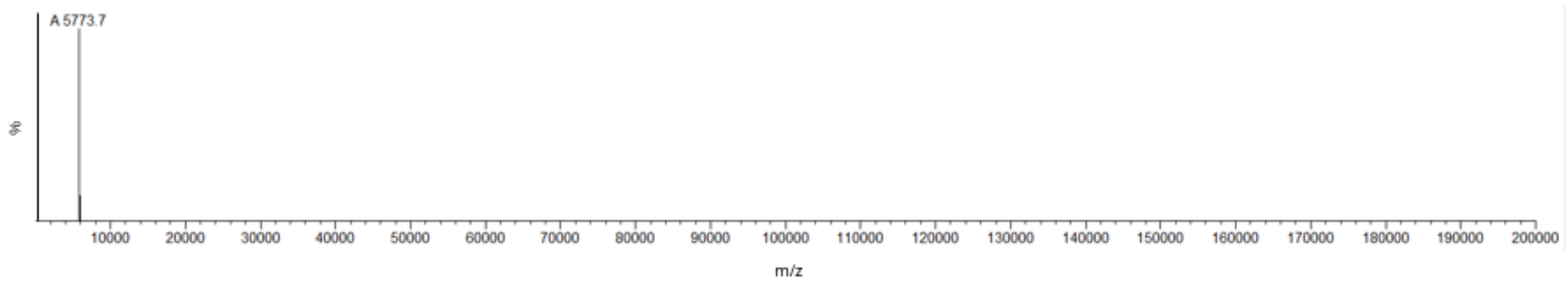

Figure S10. Mass spectrum of the final peptide (5774.0: molecule peak).

References

1. Castanar, L.; Poggetto, G. D.; Colbourne, A. A.; Morris, G. A.; Nilsson, M., The GNAT: A new tool for processing NMR data, *Magn Reson Chem* **2018,** *56* (6), 546-558. 10.1002/mrc.4717
2. Negroni, M.; Kurzbach, D., Residue-resolved monitoring of protein hyperpolarization at sub-second time resolution, *Commun Chem* **2021,** *4* (1), 147. 10.1038/s42004-021-00587-y
3. Weber, E. M. M.; Sicoli, G.; Vezin, H.; Frebourg, G.; Abergel, D.; Bodenhausen, G.; Kurzbach, D., Sample Ripening through Nanophase Separation Influences the Performance of Dynamic Nuclear Polarization, *Angew Chem Int Ed Engl* **2018,** *57* (18), 5171-5175. 10.1002/anie.201800493
4. Kress, T.; Che, K.; Epasto, L. M.; Kozak, F.; Negroni, M.; Olsen, G. L.; Selimovic, A.; Kurzbach, D., A novel sample handling system for dissolution dynamic nuclear polarization experiments, *Magnetic Resonance* **2021,** *2*, 387-394. 10.5194/mr-2-387-2021
5. Brandis, D.; Turhan, E.; Zachrdla, M.; Kurzbach, D., Residue-Resolved Liquid-State Hyperpolarized NMR of Peptide Condensate Surfaces, *J Am Chem Soc* **2025,** *147* (40), 36920-36927. 10.1021/jacs.5c15053
6. Negroni, M.; Turhan, E.; Kress, T.; Ceillier, M.; Jannin, S.; Kurzbach, D., Fremy's Salt as a Low-Persistence Hyperpolarization Agent: Efficient Dynamic Nuclear Polarization Plus Rapid Radical Scavenging, *J Am Chem Soc* **2022,** *144* (45), 20680-20686. 10.1021/jacs.2c07960
7. Schanda, P.; Kupce, E.; Brutscher, B., SOFAST-HMQC experiments for recording two-dimensional heteronuclear correlation spectra of proteins within a few seconds, *J Biomol NMR* **2005,** *33* (4), 199-211. 10.1007/s10858-005-4425-x
8. Turhan, E.; Potzl, C.; Brandis, D.; Faglia, F.; Zachrdla, M.; Kurzbach, D., Deep Learning for Hyperpolarized NMR of Intrinsically Disordered Proteins Without Resolution Loss: Access to Short-Lived Intermediates, *Chemistry* **2025**, e02067. 10.1002/chem.202502067
9. Karampas, I.; Kontoyannis, C., Characterization of calcium phosphates mixtures, *Vibrational Spectroscopy* **2013,** *64*, 126-133.
10. Chien, Y.-C.; Hincke, M.; Vali, H.; McKee, M., Ultrastructural matrix–mineral relationships in avian eggshell, and effects of osteopontin on calcite growth in vitro, *Journal of Structural Biology* **2008,** *163* (1), 84-99.
